# Sleep Disordered Breathing and Neurocognitive Disorders

**DOI:** 10.3390/jcm13175001

**Published:** 2024-08-23

**Authors:** Ikechukwu Ogbu, Tushar Menon, Vipanpreet Chahil, Amrit Kahlon, Dakshinkumaar Devanand, Dinesh K. Kalra

**Affiliations:** Department of Cardiology, University of Louisville, Louisville, KY 40202, USA

**Keywords:** cerebral small vessel disease, sleep apnea, sleep disordered breathing, cognitive impairment, dementia

## Abstract

Sleep-disordered breathing (SDB), which includes conditions such as obstructive sleep apnea (OSA) and central sleep apnea (CSA), is an independent risk factor for cerebral small vessel disease (CSVD), stroke, heart failure, arrhythmias, and other cardiovascular disorders. The influence of OSA on brain structure and cognitive function has become an essential focus in the heart-brain axis, given its potential role in developing neurocognitive abnormalities. In this review, we found that OSA plays a significant role in the cardio-neural pathway that leads to the development of cerebral small vessel disease and neurocognitive decline. Although data is still limited on this topic, understanding the critical role of OSA in the heart-brain axis could lead to the utilization of imaging modalities to simultaneously identify early signs of pathology in both organ systems based on the known OSA-driven pathological pathways that result in a disease state in both the cardiovascular and cerebrovascular systems. This narrative review aims to summarize the current link between OSA and neurocognitive disorders, cardio-neural pathophysiology, and the treatment options available for patients with OSA-related neurocognitive disorders.

## 1. Introduction

OSA is the most common form of SDB and is characterized by repeated episodes of upper airway collapse due to reduced muscle tone while sleeping. These blockages can be complete (apneas) or partial (hypopneas), leading to a cycle of intermittent low oxygen levels in the blood, disturbances in autonomic nerve function, and disrupted sleep patterns. OSA is a heterogeneous disorder characterized by different phenotypes [1,2,3,4]. These phenotypes refer to categories of patients with OSA that can be distinguished from others by a single disease feature or a combination of disease features. These phenotypical features includes symptoms, polysomnography (PSG) characteristics (hypoxemia, Apnea-Hypopnea Index (AHI)), response to therapy, comorbidities, and incidental cardiovascular disorders [1,4]. OSA is characterized by intermittent partial or complete recurrent blockage of the upper airway due to decreased muscle tone during sleep [5,6,7].

The widespread comorbidity profile of OSA, encompassing cardiovascular, metabolic, and neuropsychiatric domains, suggests complex bidirectional relationships with conditions like heart failure, stroke, and metabolic syndrome [3,5]. Recent studies have revealed distinct OSA phenotypes beyond obstruction frequency, showing links with specific symptomatic manifestations [2,3]. It is vital to bridge the gap between measurable patient characteristics, phenotypes, and underlying pathophysiological traits to enhance our understanding of OSA and its interplay with related neurocognitive outcomes [5,6,8]. However, regardless of the sleep disorder phenotypes, studies have shown that OSA is prevalent in approximately 30% to 80% of older patients who meet the criteria for some level of cognitive impairment or have a clinical diagnosis of Alzheimer’s disease (AD). Globally, over 1 billion individuals experience some form of sleep apnea, which is a significant risk factor for all-cause mortality and morbidity, especially in older patients. Meanwhile, according to the World Health Organization (WHO), in 2021, progressive neurocognitive disorders, such as dementia, affect over 50 million individuals and are the seventh leading cause of death globally [9,10,11].

The association between OSA and neurocognitive abnormalities has been linked to periodic episodes of low oxygen levels, a characteristic feature of OSA, which can trigger downstream widespread inflammation, hormonal fluctuations, and other pathophysiological changes [12,13]. These changes may result in cerebral biochemical alterations. Interestingly, some studies have shown that some of these OSA-driven alterations are biologically similar to the ones seen in neurodegenerative disorders that are genetically driven [12,13,14]. Progress in neuroimaging techniques has unveiled structural and functional modifications in the brains of individuals suffering from OSA. Notably, these modifications have been associated with reduced gray matter volume in crucial regions such as the hippocampus and frontal lobes, which play a pivotal role in memory consolidation and executive functioning [15,16,17].

Aside from neurocognitive effects, OSA is recognized as a significant independent risk factor for other severe conditions. These include heart failure, cardiac arrhythmias, stroke, pulmonary hypertension, myocardial ischemia, and myocardial infarction [14,18,19]. There is a growing body of evidence suggesting that OSA may heighten the risk of cognitive impairment or dementia through a heart-brain mechanistic link. However, evidence supporting the exact mechanism remains limited [20,21,22]. Age is an essential non-modifiable factor in the heart-brain pathomechanism. As an individual ages, the gradual increase in stiffness and pressure in the proximal aorta leads to more significant pulsation into the cerebral microvasculature, potentially leading to the onset or progression of CSVD [23,24,25,26]. Other studies have shown that CSVD has been indirectly linked to aortic stiffening, another critical disease state in the cardio-neural axis involved in the subsequent development of neurocognitive disorders in SDB patients [9,26,27].

CSVD is a type of arteriosclerosis that plays a significant role in cerebrovascular conditions. It is commonly associated with persistent microvascular alterations or changes in the white matter, especially in older adults [28]. The brain is extensively vascularized, except for the white matter regions, which are particularly susceptible to reduced blood flow [29]. Neuroimaging often reveals signs of decreased perfusion and pulsatile microvascular damage in the brain’s white matter. These signs include white matter hyperintensities (WMH), cerebral atrophy, chronic microvascular changes, expansion of perivascular spaces, and silent brain infarction (SBI) [30,31,32].

Insights into the role of SDB in the pathomechanisms leading to neurocognitive abnormalities could pave the way for new diagnostic protocols and targeted therapies. Therapies such as continuous positive airway pressure (CPAP), which prevents the collapse of the upper airway during sleep, have been shown to enhance oxygenation, decrease nighttime breathing events, alleviate daytime sleepiness, improve sleep-related quality of life, and reduce risks for major adverse cardiac events and all-cause mortality [8,33,34]. However, further research is needed to understand how treatments like CPAP therapy influence brain structure and neurocognitive impairments. This review will concentrate on the epidemiology, risk factors, and pathophysiology leading to neurocognitive abnormalities in patients with OSA, as well as its treatment. We will not discuss other SDB disorders, such as CSA, complex sleep apnea, or other sleep-related hypoxemia or hypoventilation disorders unless they are relevant to the discussion of OSA.

## 2. Materials and Methods

We conducted a comprehensive narrative review following the Preferred Reporting Items for Systematic Reviews and Meta-Analyses (PRISMA) guidelines. A comprehensive literature search was performed using the PubMed, Embase, and Web of Science databases from 2000 to 2024. The search strategy included a combination of MeSH terms and keywords related to “obstructive sleep apnea”, “neurocognitive disorders”, “cognitive impairment”, and “dementia”. The search was limited to articles published in English. Full-text articles were retrieved for further assessment if the study met the inclusion criteria: (1) original research articles, (2) studies investigating the association between OSA and neurocognitive disorders, and (3) studies providing sufficient data for analysis. The following data were extracted from the included studies: first author’s name, year of publication, study design, sample size, characteristics of the study population (age, sex, comorbidities), OSA assessment method, neurocognitive assessment method, main findings, and conclusions.

The quality of the included studies was assessed using the Newcastle-Ottawa Scale for observational studies and the Cochrane Risk of Bias Tool for randomized controlled trials. Studies were evaluated based on the selection of the study groups, the comparability of the groups, and the ascertainment of the outcome of interest. A narrative synthesis was conducted, and the results were organized based on the type of neurocognitive disorder investigated. The potential mechanisms linking OSA and neurocognitive disorders were also discussed. This methodology ensures a comprehensive and systematic approach to identifying, selecting, and synthesizing the relevant literature on the association between OSA and neurocognitive disorders. It also allows for the assessment of the quality of the included studies, which is crucial for interpreting the findings of this review.

## 3. Epidemiology and Risk Factors

Globally, progressive neurocognitive disorders, such as dementia, affect over 50 million individuals and are the seventh leading cause of death [35]. A meta-analysis by Aini revealed that nearly half, 49% (95% CI 25–73%) of dementia patients suffer from SDB [36]. Moreover, a comprehensive meta-analysis by Leng, which included more than 4 million individuals, found an increased risk of cognitive impairment in patients with SDB, with a risk ratio (RR) of 1.35 (95% CI 1.11–1.65) [37]. Other epidemiological studies have found that SDB is a prevalent respiratory condition, especially among older patients, with some studies suggesting that up to 60% of older adults may be affected [38]. Zhu and colleagues conducted a meta-analysis of 19,940 patients with baseline SDB. The study found that individuals with SDB at baseline had a significantly higher risk of cognitive decline, as indicated by a combined outcome of mild cognitive impairment (MCI) or dementia [38]. The cognitive deficits in individuals with SDB include attention and vigilance, episodic memory, long-term visual and verbal memory, visuospatial/constructional abilities, and executive functions. These deficits can be quantified through objective assessments such as the Wechsler Adult Intelligence Scale and the Psychomotor Vigilance Task [39,40].

Inpatient polysomnography (PSG) is the gold standard for diagnosing OSA and its severity. However, the presence of other sleep-related disorders such as insomnia can lead to diagnostic and therapeutic setbacks. The grading of OSA severity is based on AHI, where apnea refers to complete airflow cessation for more than 10 s, and hypopnea refers to a reduction in airflow to less than 50% of normal levels. An AHI index of 5–15 indicates mild OSA severity; an index of 16–30 indicates moderate severity; and an index greater than 30 indicates severe OSA [5,6,33,41]. With increasing life expectancy and improved survival rates after acute cardiovascular events, preserving brain health has become one of the significant challenges in modern cardiovascular medicine [5,42]. There is increasing new evidence for a causal relationship between specific risk factors and sleep disturbances, such as the REM disruption seen in OSA and the risk of developing neurocognitive abnormalities. However, the results from many studies have varied [43,44,45,46,47]. Except for anatomic structures like cranial features, OSA and neurocognitive disorders share common risk factors such as sex, age, obesity, inflammation, and genetics [20,41,48,49,50,51,52,53,54,55,56]. Table 1 shows the effect of some shared risk factors associated with OSA and cognitive decline [49,50,53,55,57,58,59,60,61,62,63,64,65,66,67,68,69,70,71,72,73,74,75,76,77].

Risk factors known to impact neurocognitive impairments include aging, sex, and genetic predispositions, such as the apolipoprotein E ε4 allele. The significant risk factors for OSA include age, obesity, smoking, chronic nasal blockage, male sex, and genetics [2,79]. Although no specific genetic factors have been conclusively associated with neurocognitive disorders related to OSA, it is widely known that genetic and non-genetic risk factors can contribute to a predisposition to OSA and neurocognitive disorders such as AD. These combined predispositions could indirectly affect cognitive outcomes [39]. Certain medical conditions can intensify the neurocognitive effects of OSA. For example, vascular changes associated with hypertension and hyperlipidemia can lead to cognitive alterations. Hormonal changes, such as alterations in insulin secretion or sensitivity, cortisol, leptin, and testosterone, can also play a significant role [39,80].

OSA and obesity metabolic syndrome are associated with sympathetic nerve activation and increased C reactive protein, interleukin-6, and tumor necrosis factor (TNF) [80]. The consumption of certain substances, particularly sedatives and alcohol, can exacerbate OSA symptoms and potentially lead to cognitive impairment. Age is another crucial factor, with OSA being more prevalent among individuals aged over 65. This age group is also more prone to cognitive decline, which could potentially amplify the impact of OSA [39,80,81].

**Figure 1:**
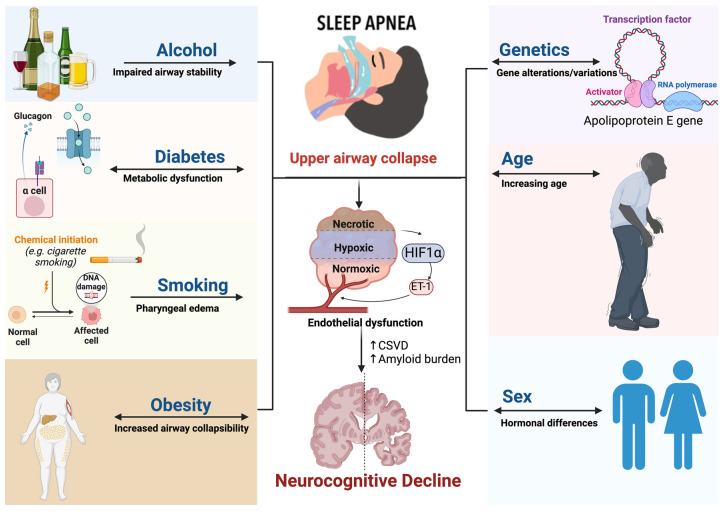
Shared risk factors between OSA and neurocognitive abnormalities. Common risk factors such as sex, age, obesity, inflammation, and genetics have shown a causal relationship between OSA and cognitive decline. Abbreviation: OSA = obstructive sleep apnea; NAD = nicotinamide adenine dinucleotide; DNA = deoxyribonucleic acid, RNA = ribonucleic acid; ET-1 = endothelin 1; HF1a = hypoxia-inducible factor 1-alpha; α cell = alpha cell; CSVD = cerebral small vessel disease.

The severity of OSA, often quantified by AHI, can influence the extent of cognitive impairment. More severe cases of OSA can result in more significant cognitive deficits [38]. In addition, several pathophysiological factors may affect cognitive function in OSA patients. These include intermittent hypoxia, systemic inflammation, and oxidative stress. Chronic inflammation, a common feature in OSA, is an additional risk factor for cognitive decline [50,80,82]. Figure 1 shows the shared risk factors between SDB and neurocognitive decline.

### 3.1. Genetics

The apolipoprotein E (ApoE) gene, particularly its variant ApoE4, is strongly linked to the emergence of AD, especially the early-onset form. This variant is the most prevalent and powerful genetic risk factor, accounting for more than half of all AD instances [83,84]. Roughly one in four people possess a single copy of ApoE4, while 2% to 3% have two copies [85]. However, the existence of ApoE4 does not necessarily lead to the disease. Multiple risk factors, such as other genetic components, lifestyle habits, and environmental influences often determine individual health outcomes. However, the ApoE4 variant plays a crucial role in Alzheimer’s Disease, and its presence can influence the speed of neurocognitive deterioration in patients with SDB [85,86,87].

In some studies, the ApoE4 variant has been linked to sleep disturbances in healthy older adults and an increased risk of insomnia [88]. Studies have also found that SDB patients with Alzheimer’s disease who carry the ApoE4 variant experience neurocognitive decline at a faster pace than those without the variant [88,89]. This suggests that ApoE4 may influence sleep through different pathomechanisms distinct from the pathological changes observed in Alzheimer’s disease patients with SDB [88].

### 3.2. Age

SDB is common in older people and is often unrecognized and undiagnosed. Studies have found that SDB in older people is associated with worsening cardio-cerebrovascular, cognitive, and functional outcomes [81]. A Multi-Ethnic Study of Atherosclerosis (MESA) involved 622 participants and examined the association of epigenetic age acceleration using DNA methylation (DNAm), a biomarker of fast biological aging [82]. In the MESA study, AHI was associated with greater DNAm-PhenoAge acceleration (b = 0.03; 95% CI [0.001, 0.06]). The arousal index was associated with greater DNAm-Age acceleration (b = 0.04; 95% CI [0.01, 0.07]). Both associations were stronger in women than in men [82].

A prospective multicenter cohort study by Redline and colleagues examined the relationship between age progression and SDB in 2685 patients [2]. This study used polysomnography to measure various parameters related to sleep quality, including airflow, oximetry, thoracic/abdominal excursions, electrocardiogram, and heart rate. The study also compared trends between common risk factors and the impact of SDB on sleep architecture [2]. The study found that as age increased, sleep architecture deteriorated. This deterioration was characterized by a lower percentage of rapid eye movement (REM) sleep and an increased arousal index (ARI), which is the approximate number of times a patient wakes up per hour of sleep [2]. These results were thought to be due to age-related physiological changes in hormone release from the somatotropic axis. These changes increased ARI and respiratory disturbance indices (RDI) during sleep, thereby increasing the risk of SDB [2,69,81].

Another study by Fietze and colleagues focused on the impact of age differences on OSA. The study involved 1208 participants aged between 20 and 81 [69]. The participants completed questionnaires/interviews and underwent nine days of PSG examinations. The AHI values from the PSG data were used to categorize OSA severity. An AHI score of ≥5 was classified as mild to moderate OSA, and an AHI score of ≥15 was classified as moderate to severe OSA. The study found significant elevations in AHI scores among men older than 70 years of age, with scores as high as 15.2 (95% CI 11.7–18.7) [69]. For women, AHI scores began to significantly elevate from baseline at age 50 [69]. The study also reported significant odds ratios between age and AHI ≥ 15. The odds ratio was 1.94 (95% CI 1.66–2.27) for men and 3.20 (95% CI 2.41–4.25) for women [69]. This suggests that age and sex are significant factors in the severity of OSA.

### 3.3. Sex

OSA is more prevalent in men than in women. Studies have shown that the estimated prevalence of OSA ranges from 13% to 33% in adult men and 6% to 19% in adult women [6,72,90]. This discrepancy has been attributed to differences in body fat distribution, specifically increased upper airway fat distribution in men and pharyngeal anatomy and function [6,90]. A study found that 55% of OSA patients have a prominent increase in obstructive apneic events (OAEs) during NREM sleep stages compared to REM sleep stages [91]. In that study, men were at a higher risk for OSA during non-rapid eye movement (NREM) sleep [91]. At the same time, another study found that men typically exhibit more severe OSA during NREM sleep [92].

On the other hand, both men and women have been found to have a similar risk of OSA during REM sleep [93]. Other studies have found that the prevalence of REM-related OSA is higher in women than in men [94,95]. This suggests that while men are more prone to OSA during NREM sleep, the risk during REM sleep is similar for both sexes, with potentially higher prevalence among women. This highlights the complex interplay of factors contributing to the risk and prevalence of OSA across different sleep stages and sex.

The connection between OSA and cognitive decline appears to exhibit sex-based differences, with age appearing to influence this discrepancy [87,96]. Current research suggests a potential sex bias in the occurrence of dementia among OSA patients [49]. A study found that women diagnosed with OSA had a higher likelihood of developing dementia compared to their age- and sex-matched counterparts who did not have OSA [87]. This suggests that the risk of cognitive impairment could be more significant in women with OSA than in men [49,87]. The exact reasons for these sex differences remain unclear. However, it has been proposed that the impact of sex hormones on development—which leads to sexual differentiation—might render the female brain more susceptible to Alzheimer’s disease pathology [49]. Findings from these studies suggest that the prevalence of OSA is higher in men than in women; however, women with OSA appear to have an increased risk of cognitive impairment and dementia. This difference seems to be age-dependent and is likely the result of a complex interaction of biological, hormonal, and possibly environmental factors. However, additional research is required to understand the underlying mechanism.

### 3.4. Alcohol

In a prospective cohort study that examined incidental dementia in over 9000 patients with a mean follow-up of 23 years, among those drinking more than 14 units of alcohol per week, a 7-unit increase in consumption was associated with a 17% (95% CI: 4–32%) increase in the risk of dementia [75]. A randomized controlled trial by Koch and colleagues showed that among participants with MCI, the hazard ratio for dementia was 1.72 (95% CI, 0.87–3.40) for those consuming more than 14.0 drinks per week compared with those consuming less than 1.0 drinks per week. Findings were consistent when stratified by sex, age, and APOE ε4 genotype [76].

The ingestion of alcohol before sleep can influence the sleep cycle and the quality of deep sleep by modifying sleep patterns and stages [79]. Alcohol can induce an increase in N3 sleep, often referred to as “deep sleep”, and a decrease in REM sleep [79]. As the night progresses and the body processes the alcohol, there is a likely increase in N1 sleep, the lightest sleep stage. This can result in frequent awakenings and disrupted, poor sleep quality [79,97]. Moreover, alcohol consumption can intensify OSA due to airway instability. As a relaxant, alcohol can aggravate airway obstruction in sleep apnea. An individual with OSA is susceptible to more frequent and prolonged breathing interruptions after consuming alcohol. Numerous studies have demonstrated that alcohol usage elevates a person’s AHI, which quantifies the number of times per hour a person’s breathing is paused or restricted. This can decrease blood oxygen levels, potentially leading to an increased risk of severe cardiocerebrovascular complications from OSA [79,97].

The role of alcohol consumption in the onset of various neurocognitive conditions linked to OSA has been the subject of extensive research. A case-control study that included 793 participants, 688 of whom were OSA patients, and 106 who were control subjects, explored the connection between alcohol consumption and OSA [73]. The study revealed a significant association between alcohol consumption and an increased risk for OSA, with a notable odds ratio of 2.03 (95% CI 1.30–3.17) and odds ratios of 1.96 (95% CI 1.19–3.22, *p* < 0.05) and 2.22 (95% CI 1.06–4.63, *p* < 0.05) after accounting for past and present drinkers [73]. The study also discovered a positive correlation between AHI values and alcohol consumption, and link between chronic alcohol usage and damage to alveolar type 2 cells, which could potentially lead to upper airway collapse in OSA patients [73]. A meta-analysis that investigated the relationship between alcohol consumption and OSA found that men with OSA who regularly consumed alcohol were 1.33 times more likely to have an AHI greater than 15 events/hour compared to men who abstained from alcohol [78]. However, no significant correlations were found between women with OSA and alcohol consumption [78].

### 3.5. Smoking

In a prospective multiethnic study of over 21,000 patients, a total of 5367 people (25.4%) developed dementia after adjusting for age, sex, education, race, marital status, hypertension, hyperlipidemia, body mass index, diabetes, heart disease, stroke, and alcohol use. The study found that smoking more than two packs of cigarettes a day was associated with a significant risk of dementia (adjusted hazard ratio [HR], 2.14; 95% CI, 1.65–2.78), Alzheimer’s disease (adjusted HR, 2.57; 95% CI, 1.63–4.03), and vascular dementia (adjusted HR, 2.72; 95% CI, 1.20–6.18) [77]. The results of this study suggest that smoking could promote oxidative stress and neuroinflammation that drive the development of neurocognitive decline [77].

Other studies have also found a link between smoking and snoring, an initial symptom of OSA [74,98]. The inhalation of harmful substances in cigarette smoke, such as pollutants and chemicals, can lead to airway inflammation and increase the likelihood of airway collapse [99]. Furthermore, nicotine, a stimulant in tobacco, can disrupt sleep architecture. This disruption can cause challenges in falling asleep or staying asleep. It also modifies the transition between different sleep stages, which decreases time spent in deep, restorative sleep [74,99]. Poor sleep quality can significantly impact neurocognitive function. Studies examining the effects of sleep on neurocognitive functioning have broadly found that inadequate sleep and daytime sleepiness manifest in poorer performance on tasks involving attention, concentration, memory, reasoning, and thinking [74,99]. Short-term daytime cognitive impairment is common in people with sleep deprivation, insomnia, sleep apnea, or other conditions that prevent them from getting adequate rest. Over the long term, poor sleep may put someone at a higher risk of cognitive decline and dementia [74,99].

A comprehensive review found that individuals who smoke, especially heavy smokers with a history of smoking more than 20 pack per year are at an elevated risk of OSA [100]. The review also discovered that severe OSA patients had a significant correlation with smoking compared to those with mild or moderate OSA [100].

### 3.6. Diabetes and Obesity

Obesity is a key moderator of the effect of OSA on type 2 diabetes. The research by Young and colleagues highlights the significant role of obesity in OSA. They estimated that approximately 17% of adults have OSA, with 41% to 58% of mild-to-moderate OSA cases can be attributable to excess weight [64]. This is consistent with population-based studies from various countries, including Spain, Italy, Brazil, India, Korea, and Australia, which confirm obesity as one of the strongest predictors of OSA globally [101]. OSA alters glucose metabolism, promotes insulin resistance, and is associated with the development of type 2 diabetes. Type 2 diabetes is characterized by insulin resistance and relative insulin deficiency, leading to elevated blood glucose levels. Complications from type 2 diabetes can include cardiovascular disease, nerve damage, kidney damage, and other neurovascular and neurocognitive complications [102,103]. A study by Tasali and colleagues contributes to understanding the relationship between type 2 diabetes and OSA. The research suggests that OSA may affect glucose metabolism and could be a risk factor for developing type 2 diabetes [103].

Research conducted within a community setting found that men with type 2 diabetes have a 23% prevalence rate of OSA, a rate notably higher than the 6% seen in the broader population [103]. A separate multicentric, cross-sectional study revealed a high rate of undiagnosed OSA among obese individuals with type 2 diabetes. PSG showed that approximately 75% of these patients had moderate-to-severe OSA [104]. Given the established complications associated with both diabetes and OSA and their impact on neurocognition, this prevalence suggests that type 2 diabetes and OSA could jointly exacerbate cognitive health issues. OSA is common among patients with type 2 diabetes [57]. Chronic sleep fragmentation, sleep deprivation, and intermittent nocturnal hypoxemia associated with OSA have been implicated in metabolic dysfunction, including altered glucose metabolism [57]. Increasing evidence suggests a link between SDB and type 2 diabetes, glucose intolerance, and insulin resistance. In the Sleep Heart Health Study, OSA was associated with impaired fasting glucose (IFG), impaired glucose tolerance (IGT), and occult diabetes after adjusting for age, sex, race, BMI, and waist circumference [57]. This correlation is especially concerning, considering that OSA often goes undetected. Individuals suffering from OSA experience sleep disruption, which, when coupled with the neurovascular complications of type 2 diabetes, can significantly decrease a person’s quality of life. These findings underscore the urgent need for increased awareness and screening for OSA among type 2 diabetes patients exhibiting neurocognitive irregularities, particularly those who are obese [58,103,105].

## 4. Pathophysiology

Several pathomechanisms link OSA-related sleep disturbances with neurocognitive abnormalities [106]. A study by Pase and colleagues examined sleep architecture and its association with adult cognitive function. The study highlighted the significant impact of sleep disturbances on cognitive health, with mild to severe OSA associated with poorer global cognition (pooled β, −0.06; 95% CI, −0.11 to −0.01; *p* = 0.01) versus AHI less than 5; comparable results were also found for moderate to severe OSA (pooled β, −0.06; 95% CI, −0.11 to −0.01; *p* = 0.02) versus AHI less than 5 [106]. Another study by Seda and colleagues examined the cognitive effects of untreated OSA and the benefits of treatment across cognitive domains [107]. The study found that OSA’s greatest impact appears to be on attention, vigilance, and information processing speed. Furthermore, the presence of OSA seems to impact the development and progression of MCI significantly [107].

### 4.1. Role of Hypoxia and Endothelial Dysfunction

The repetitive episodes of upper airway obstruction lead to intermittent hypoxia and reoxygenation [108]. This process, known as hypoxia and reperfusion injury, can lead to higher lipid peroxidation [13,109,110]. Lipid peroxidation produces higher levels of soluble adhesion molecules and reactive oxygen species, which can harm the brain [79,111,112]. Endothelial dysfunction, another consequence of OSA, can cause an imbalance between vasoconstriction and vasodilation, promoting hypercoagulability and lead to atherosclerosis via plaque deposition [113]. OSA has been found to decrease nitric oxide (NO) levels, a potent vasodilator, thus resulting in a mismatch between vasoconstriction and vasodilation [113,114,115].

Endothelial cells in the vascular system preserve the equilibrium between vasoconstriction and vasodilation processes and control various pro-inflammatory and anti-inflammatory markers [113]. OSA can lead to pathological dysfunction of these endothelial cells, disrupting this balance. OSA is recognized as a condition of persistent inflammation, which can eventually cause irreversible alterations that contribute to a decline in neurocognitive function [113,116,117]. Repetitive episodes of low oxygen seen in OSA patients are associated with the upregulation of inflammatory markers, such as an increase in the production of C-reactive protein (CRP), soluble adhesion molecules, and leukocyte peroxide, and downregulation of anti-inflammatory markers [113,117]. Vascular inflammation in OSA leads to increased levels of COX-2, a potent inflammatory protein. The upregulation of COX-2 leads to increased superoxide production, resulting in oxidative stress, platelet activation, endothelial dysfunction, and vasoconstriction [56,113,116,117]. OSA results in higher levels of inflammatory markers, including IL-4, and downregulation of the anti-inflammatory cytokine IL-10. This propagates the inflammatory cascade, further developing endothelial dysfunction [113,117].

Other studies have found that the severity and number of these hypoxic events positively correlate with the extent of the associated neurocognitive pathology [118,119]. Due to cyclic apnea and hypopnea episodes, hypoxia increases endothelin-1 levels and causes vasoconstriction, enhanced expression of adhesion molecules, and amyloid fibril formation, which develops into senile plaques [118]. These plaque accumulations cause neurotoxicity and induce tau pathology, leading to neuronal cell death and neurodegeneration [118]. Moreover, OSA-induced intermittent hypoxia results in hyperpermeability of the blood-brain barrier (BBB) and neuroinflammation, leading to altered synaptic plasticity, neuronal damage, and worsening of cerebral small vessel disease (CSVD) [56,113,118,119]. These findings suggest that OSA may play a vital role in the early pathological processes of CSVD and BBB dysfunction, resulting in the development or exacerbation of neurocognitive deficits or dementia.

### 4.2. Role of Pulse Wave Velocity and Cerebral Pulsatility

Current evidence reveals a negative association between arterial stiffness, measured using pulse-wave velocity (PWV), and cognition, specifically executive function, memory, and global cognition. This association appears independent of demographic, clinical, and assessment characteristics [120]. PWV, a measure of arterial stiffness, is known to increase with each decade of life. This increase in PWV is associated with cardiovascular and cerebrovascular diseases, as the endothelium becomes calcified due to atherosclerotic plaque deposition, leading to a decrease in elasticity and an increase in arterial stiffness [121]. This is further supported by studies suggesting that an increase in PWV is associated with higher amyloid-beta (Aβ) plaque deposition in the brain, which can ultimately lead to the development of symptomatic dementia [121,122,123].

**Figure 2:**
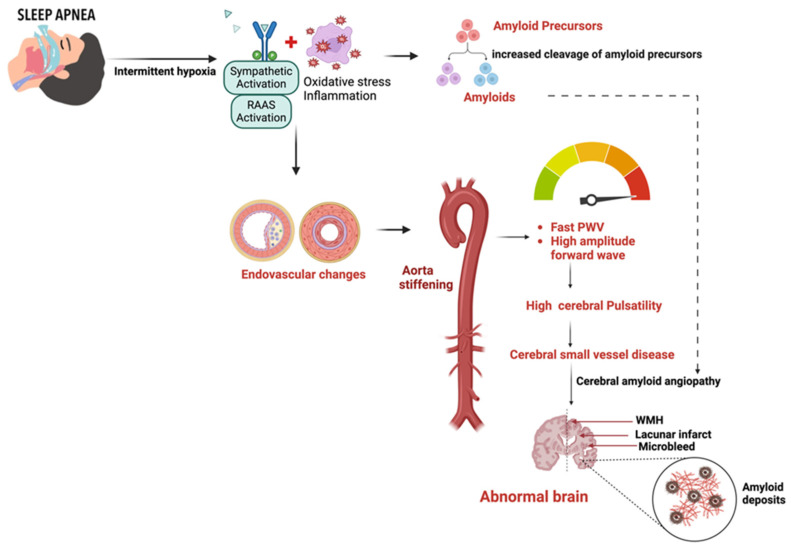
Prolonged exposure of the cerebral circulation to intermittent hypoxia and higher pressure leads to endothelial remodeling, neuroinflammation, ischemic, hemorrhagic injury, and cognitive impairment. Abbreviation: PWV = pulse wave velocity; WMH = White Matter Hyperintensities; RAAS = renin-angiotensin-aldosterone system.

Aside from cerebral autoregulation, the proximal aorta in the heart-brain connection is a vital coupling device that dampens the high-pressure waveform from the left ventricle to the cerebral vascular beds [29,34,120,124,125]. Therefore, increased arterial stiffness in the aorta can lead to consequential cerebrovascular pathology due to an impaired regulatory buffer between heart and brain perfusion, which regulates the pressure and flow pulsatility transmitted into the brain microcirculation [29,124,126,127,128]. As aortic stiffness increases, the higher pulsatility, if transmitted into microcirculation, could potentially damage small vessels with low resistance [29,129,130]. A chronic dysregulation in the heart-brain link can enhance the pathophysiological cascade that contributes to cerebral small vessel disease, leading to cognitive decline that eventually advances to dementia [Figure 2].

The relationship between arterial stiffness, quantified by pulse pressure (PP) and pulse wave velocity (PWV), and cognitive decline highlights the role of vascular factors in neurocognitive disorders in SDB [131]. The Baltimore Longitudinal Study of Aging involved 1749 participants for PP and 582 for PWV analyses, all stroke-free and non-demented at baseline. Participants were assessed over 14 years, with cognitive function evaluated across multiple domains, including verbal and nonverbal memory, attention, perceptuo-motor speed, and executive functions [131]. The study demonstrated that higher baseline PP and PWV predicted accelerated cognitive decline, particularly in verbal memory (*p* = 0.001 for California Verbal Learning Test (CVLT) learning slope and *p* = 0.003 for CVLT long delay free recall), nonverbal memory (*p* = 0.0001 for Benton Visual Retention Test (BVRT)) and working memory (*p* = 0.043 for Digits Backward). This acceleration in cognitive decline can be attributed to arterial stiffness, which exacerbates neurodegenerative processes by increasing cerebral pulsatile load, leading to microvascular damage and impaired cerebral perfusion [131].

Population studies have also shown that arterial stiffening is linked to white matter hypersensitivity, cerebral atrophy, and cognitive impairment, all of which are features of cerebral small vessel disease [28,131,132,133]. The pathophysiological link between the role of OSA in endothelial dysfunction could offer valuable insights into the mechanism that predisposes a low-resistance cerebral microvascular bed to small vessel diseases that lead to impaired beta-amyloid and tau protein clearance and results in cerebral amyloid angiopathy [29,36,134,135,136]. The impact of such SDB-driven hemodynamic factors could result in an indirect SDB-induced increase in cerebral pulsatility index (PI) because of upstream factors such as increased aortic stiffness and pressures [128,137].

**Figure 3:**
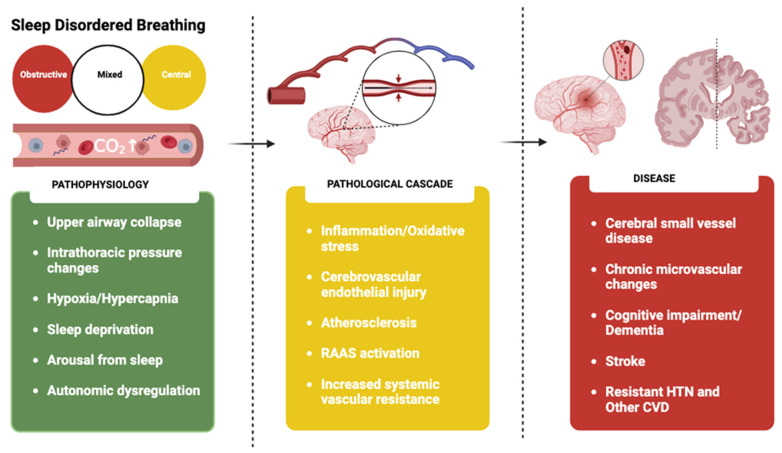
The pathophysiological cascade linking SDB with neurocognitive abnormalities. OSA leads to endothelial remodeling, neuroinflammation, disruption of the blood brain barrier, and reduced cerebrovascular compliance, which makes the brain vulnerable not only to ischemic but also to hemorrhagic injury and cognitive impairment. SDB = sleep-disordered breathing; HTN = hypertension; CO_2_ = carbon dioxide; RAAS = renin-angiotensin-aldosterone system; CVD = cardiovascular disease.

This pathophysiological mechanism might underscore the relationship between SDB and cerebrovascular events. In some studies, higher PI and aortic pulse wave velocity (PWV) were the strongest physiological correlates of WMH severity in patients diagnosed with a transient ischemic attack or minor stroke, regardless of age [128,138]. The development of WMH has been linked to chronic ischemia or amyloid microangiopathy from endothelial dysfunction. Over time, the prolonged exposure of the cerebral circulation to higher pressure can lead to endothelial remodeling, neuroinflammation, disruption of the BBB, and reduced cerebrovascular compliance, making the brain vulnerable to ischemic and hemorrhagic injury and cognitive impairment [30,50,111,138]. Figure 3 shows the pathophysiological cascade linking sleep-disordered breathing (SDB) to neurocognitive abnormalities.

### 4.3. Role of Cerebral Small Vessel Disease

Several studies have identified OSA and hypertension as independent risk factors not only for the development of cerebrovascular disease but also for worse outcomes, exacerbations, and repeat cardiovascular events [14,18,139,140,141]. In the discussion of neurocognitive abnormalities, OSA and hypertension are known to trigger cerebral hypoperfusion and glucose hypometabolism [116,142]. These conditions, along with aging, hypoxia, inflammation, and oxidative stress, synergistically promote diverse pathological mechanisms, including cerebral hypoperfusion and glucose hypometabolism, which precede neurocognitive decline [56,116,142].

Intermittent cerebral hypoxia, which can result from variations in cerebral blood flow velocity (CBFV), has been linked to increased reactive oxygen species (ROS) in neuronal cells of the hippocampal and brainstem regions [55,116,117,142]. This pathomechanism has a significant impact, because the brain’s storage capacity for oxygen and substrates is limited, making the proper regulation of cerebral blood flow (CBF) essential to meet its high metabolic needs. On a biomolecular level, an increase in ROS can initiate a series of events leading to apoptosis [112,142]. This apoptotic process relies on the excessive stimulation of N-methyl-D-aspartate receptors (NMDARs), which results in the abnormal activation of redox-mediated events. These events, in turn, activate downstream pathways that contribute to the promotion of CSVD, atrophy of the hippocampus, and an increase in amyloid-beta (Aβ), hyperphosphorylation of tau, and synaptic dysfunction [28,117,142,143].

A meta-analysis involving 27,952 participants showed a significant correlation between elevated arterial stiffness and increased CSVD burden, even after adjusting for comorbidities (OR:1.24; *p* < 0.01) [124]. Notably, increased arterial stiffness is strongly associated with the progression of white matter hyperintensities (OR:1.42; *p* < 0.01) [124]. These findings highlight the important role of arterial stiffness in developing CSVD. Increased arterial stiffness facilitates the development of CSVD and predicts its severity and progression, which can lead to significant cerebrovascular events [124]. Other studies have consistently shown that not only are patients with stroke or transient ischemic attack (TIA) more likely to have OSA, but CSVD accounts for 25% of ischemic strokes, the majority of hemorrhagic strokes, and at least 40% of dementias [8,30,141,144]. Table 2 shows recent meta-analyses on the association between SDB and neurocognitive dysfunction.

**Figure 4:**
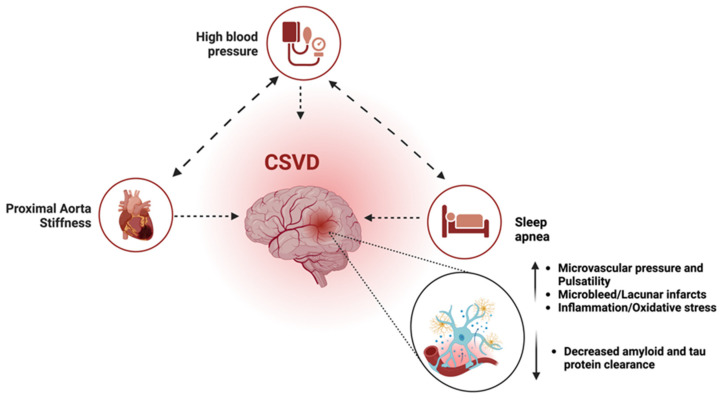
The ‘triple-hit’ effect of OSA, hypertension, and arterial stiffening on cognition. The combined effect of OSA, hypertension, and proximal aorta stiffening could propagate a triple-hit impact on cerebral microvascular beds, which potentiates the accelerated development of CSVD and amyloid build-up, the early vascular risk factors for subsequent neurocognitive abnormalities. Abbreviation: CSVD = cerebral small vessel disease; OSA = obstructive sleep apnea.

Another study investigated the impact of OSA, hypertension, and their association with arterial stiffness and heart structure [26]. The study found that severe OSA and hypertension are associated with increased arterial stiffness and heart structure abnormalities of similar magnitude, with additive effects when both conditions coexist [26]. The combined effect of OSA, hypertension, and aortic stiffening could propagate a triple-hit impact on cerebral microvascular beds, which potentiates the accelerated development of CSVD and amyloid build-up, the early vascular risk factors for subsequent neurocognitive abnormalities [37,70,127,135,145]. Figure 4 shows the combined triple effect of OSA, hypertension, and aortic stiffness on neurocognitive decline.

**Table 2:** Recent meta-analyses on the association between SDB and neurocognitive dysfunction. Several studies have shown that individuals with sleep disturbances, including poor sleep quality, circadian rhythm abnormality, insomnia, and OSA, have higher rates of cognitive decline and AD compared to individuals without sleep problems. Abbreviations: RR = relative risk, HR = hazard ratio, MCI = mild cognitive impairment, OSA = obstructive sleep apnea.

Authors	Year	Number of Studies	MCI/Dementia
Bubu [23]	2017	27	RR 1.68 (1.51–1.87)
Leng [37]	2017	14	RR 1.35 (1.11–1.65)
Shi [46]	2018	18	RR 1.19 (1.11–1.29)
Tian [70]	2023	15	HR 1.52 (1.32–1.74)
Guay-Gagnon [146]	2022	11	HR 1.43 (1.26–1.62)

### 4.4. Role of Cerebrovascular Hemodynamics

The brain’s oxygen demand requires an uninterrupted oxygen supply through cerebral circulation. This circulation is well-regulated and relies on cerebral autoregulation, cardiac output, aortic pressure gradient, and pulsatility [29,124,147]. Even briefly, OSA can cause interruptions in oxygen delivery, leading to significant pathological outcomes. This hypoxic condition triggers a pro-inflammatory state, vasoconstriction increased sympathetic activity, and the activation of chemoreceptors associated with the renin-angiotensin-aldosterone system (RAAS) [5,124,147,148].

These pathological processes also result in increased systemic vascular resistance, damage to the endothelium, elevated intracranial venous hypertension, and cerebrovascular dysfunction [19,43,149,150,151]. Chronic cerebrovascular dysregulation leads to vulnerability in cerebrovascular hemodynamics during apneic events [142,152]. Studies have shown that cerebral blood flow velocity (CBFV) changes and vascular compliance are altered in patients with severe OSA [142,153]. CBFV reactivity is significantly diminished during consecutive respiratory events, indicating that cerebral autoregulation may be insufficient to protect the brain from the rapid pressure changes seen in OSA [142,153]. Vasoneuronal coupling, which occurs due to CBFV variations during neuronal stimulation, may be suboptimal due to significant neuronal dysfunction in OSA patients [116,153]. This could enhance CBFV abnormalities and contribute to cerebral hypoperfusion [153]. Cerebral hypoperfusion may also occur due to the combination of systemic hemodynamic failure above the vasoregulatory capacity and chronic dysfunctional cerebrovascular autoregulation [153].

OSA-induced increased vascular resistance has been linked to the development of systemic hypertension, which has been linked to apnea-induced sympathoexcitation, oxidative stress, and endothelial dysfunction [154,155]. There appears to be a dose-response relationship between the severity of OSA and the odds ratio for the development of systemic hypertension, which is responsible for many cardiovascular and cerebrovascular consequences [54,156,157,158]. The proximal aorta becomes prone to arterial stiffness in systemic hypertension and increasing age [125,129,130,147]. This results in the impaired ability of the proximal aorta to act as a buffer between heart and brain perfusion, an important coupling device that regulates the amount of pressure and flow pulsatility transmitted into the brain microcirculation [29,124,126,127,128]. Impaired pressure regulation at the aortal level could damage small vessels with low resistance and drive a pathophysiological cascade, contributing to cerebral small vessel disease and amyloid buildup [121,152,153].

### 4.5. Role of Cerebral Amyloid Microangiopathy

**Figure 5:**
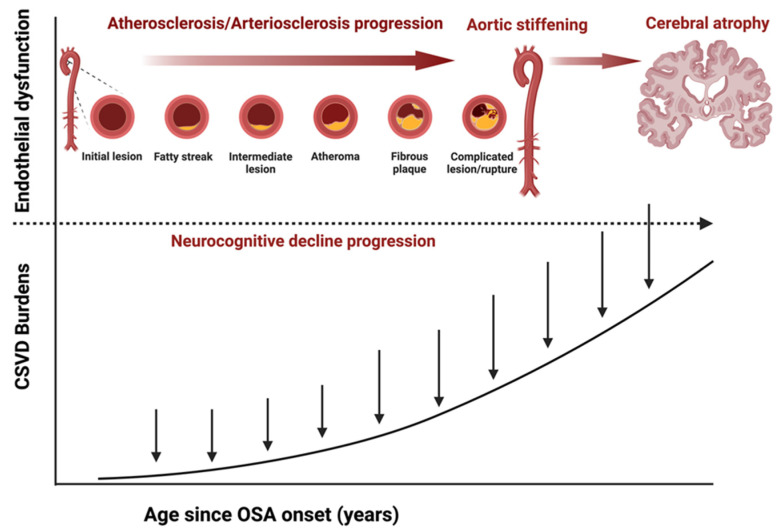
The multilayered pathophysiology and temporality of CSVD impact neurocognitive disorder. Increased arterial stiffness facilitates the development of CSVD and increases the risk of cerebrovascular events. Studies have shown that CSVD accounts for most ischemic strokes and dementias. Abbreviation: CSVD = cerebral small vessel disease.

Some research on the brain has found a correlation between OSA and an increase in amyloid burden, potentially indicating an early pathomechanism in the development of neuronal damage and neurocognitive abnormalities. Amyloid burden refers to the accumulation of amyloid-beta (Aβ) proteins in the brain, a hallmark of Alzheimer’s disease [159]. Current evidence suggests cerebral dysregulation is associated with increased cerebral amyloid burdens in patients with OSA due to endothelial stiffness in cerebrovasculature and decreased sleep-based clearance mechanisms [119,159,160]. Endothelial stiffness is a term used to describe the diminished flexibility in the lining of blood vessels. This condition can interfere with the balance within the vascular system, potentially causing vascular damage and leading to metabolic disorders and diseased states in organs such as the heart and brain. The combined role of OSA-induced sleep disruptions and impaired vascular compliance could hinder the sleep-dependent clearance processes crucial for eliminating brain waste products during sleep [119,159,160]. Figure 5 depicts the progressive vascular sequences and the temporal impact of CSVD burden leading to neuropathological conditions observed in patients with SDB.

The occurrence of CSVD and cerebral amyloid microangiopathy in individuals with OSA is believed to be associated with continuous oxygen desaturation, abnormal cerebral response to intermittent changes in oxygen and carbon dioxide levels, and cerebrovascular dysregulation are the potential pathophysiology linking OSA with neurocognitive disorders [38,43,161]. Research suggests that intermittent hypoperfusion and hypoxia caused by OSA may trigger disordered vascular remodeling and endothelial dysfunction. This can lead to arterial stiffness, reduced vascular compliance, oxidative stress, damage to the blood-brain barrier (BBB), and hindered clearance of cerebral waste, including amyloid-beta and tau proteins [22,26,134,153,162,163].

Several studies have found that CSVD accounts for approximately 40% of ischemic strokes and that stroke patients tend to have a higher burden of CSVD. It is frequently a risk factor for recurrent stroke, resulting in increased mortality, morbidity, and progressive neurocognitive impairment [9,28,153,164,165]. Some studies suggest that an AHI greater than 15, indicative of moderate sleep apnea at baseline and six months after a cerebrovascular event, is associated with a higher CSVD score [44,161]. Research has also attempted to draw a link between the severity of hypoxia and an increased accumulation of amyloid. This accumulation was attributed to an increased cleavage of amyloid precursor proteins, which leads to an increase in cerebral build-up and cerebral amyloid angiopathy [162,165,166].

Other studies have shown significant differences in amyloid burdens between untreated OSA patients and those without OSA. In OSA patients, these clearance mechanisms were shown to be utilized less frequently due to disordered sleeping patterns [15,159,160,166]. Studies have shown that OSA patients were associated with significantly higher cortical amyloid levels primarily caused by reduced sleep states and decreased activity in sleep-based clearance mechanisms. This suggests that the disrupted sleep patterns common in OSA may contribute to an increase in amyloid burdens, potentially increasing the risk of neurodegenerative diseases like AD. The link between cerebral hemodynamic dysregulation, endothelial stiffness, sleep-based clearance mechanisms, and increased cerebral amyloid burdens in OSA patients highlights the critical role of sleep in developing neurocognitive abnormalities. Table 2 shows a meta-analysis linking SDB with neurocognitive decline.

## 5. Imaging Marker of Early Cardiocerebrovascular Disease

Understanding the heart-brain link in the context of sleep apnea is crucial as it reveals how disturbances in sleep influence cerebrovascular and cardiovascular health. The combination of diagnostic tools and epidemiological studies has broadened the knowledge of the intricate heart-brain link and the relationship between aortic stiffness and neurovascular changes, increasing the risk for neurocognitive disorders [126,127]. Four established markers are used in neuroimaging to evaluate the severity of CSVD, including lacunes, white matter hyperintensities (WMH), cerebral microbleeds (CMBs), and perivascular space changes [32,126,146,165]. Evidence suggests a positive relationship between moderate to severe sleep apnea and WMH, with an OR:2.23 (95% CI 1.53–3.25). A similar relationship exists with silent brain infarcts (SBI), with an OR:1.54 (95% CI of 1.06–2.23). However, no such relationship has been observed for CMBs or perivascular spaces (PVS) [152]. This implies that untreated chronic OSA and the severity of hypoxia associated with SDB may contribute to the onset of CSVD [9,45,152]. In one study, increased AHI correlated with greater WMH volumes; specifically, for every unit increase in AHI, total WMH volume increased by 0.008 units (*p* = 0.020) and subcortical WMH volume by 0.015 units (*p* < 0.001) [153]. Additionally, higher AHI is associated with increased ePVS scores (*p* = 0.026). This link between AHI and vascular brain changes reflects the chronic effects of intermittent hypoxia and disrupted sleep architecture on cerebrovascular integrity [153].

Advancements in MRI technologies, such as susceptibility-weighted imaging and blood oxygenation level-dependent (BOLD) contrast, give us insight into these vascular changes. Susceptibility-weighted imaging enhances the visualization of venous structures and microhemorrhages by leveraging differences in magnetic susceptibility between tissues, providing insights into cerebral venous oxygenation and the microvascular alterations that promote the development and progression of small vessel diseases in the heart-brain axis [167]. The study by De Roos and colleagues showed that elevated aortic PWV, a proxy for aortic stiffness, correlates significantly with increased risks of cognitive decline and dementia [126]. MRI-based techniques, such as measuring aortic arch PWV and distensibility, further our understanding by linking aortic stiffness with CSVD progression, evidenced by white matter hyperintensities and microbleeds [126,167]. These findings reveal that pathophysiological mechanisms such as increased aortic stiffness contribute to higher cerebral microvascular pulsatility, accelerating CSVD development and impacting cognitive health [167].

The pathomechanistic heart-brain link highlighted in several studies might underscore the potential role of a heart-brain MRI protocol, which will simultaneously assess the early markers of disease processes in both organ systems. In some studies, some changes observed in neuroimaging patients with SDB have been linked to cardiovascular changes observed in cardiac MRI [9,23,162,168]. Current evidence from such studies has found that signs of cardiac changes due to hypertension—such as aortic stiffness, PWV, and the ratio of left ventricular mass to volume—are associated with cognitive decline and CSVD [28,32,127,130,131,168].

Progress in cardiac imaging technologies, particularly cardiac MRI, has significantly enhanced our ability to identify a range of heart changes and early indicators of cardiovascular diseases. Likewise, advancements in imaging of brain vessels have bolstered our capacity to detect alterations in the brain’s small vessels, which could signal the onset of cognitive decline and deteriorating brain health. However, more research is needed to determine the potential of a combined heart-brain MRI approach as an early cardiocerebrovascular disease detection strategy.

## 6. Treatment

There is compelling evidence that OSA is a modifiable risk factor for neurocognitive decline and dementia [169]. The mainstay treatment for OSA is CPAP, and multiple studies have demonstrated its effectiveness in improving neurocognitive impairment. A randomized study found that a 3-month trial of CPAP improved short-term memory, working memory, selective attention, and executive functions [169]. One hypothesis suggests that OSA might be a preclinical stage of dementia based on the result of a case study. In that case study, patients who underwent treatment with 1 year of CPAP showed normalization of CSF Aβ42 after undergoing lumbar puncture [22]. Aβ42 is a predominant biomarker shown to have a strong correlation with the pathogenesis of AD [22,170].

Research conducted by Costa and colleagues focused on the impact of CPAP therapy on cognitive abilities in a group of 171 individuals suffering from OSA and cognitive deficits due to neurodegenerative or vascular issues [171]. The study found that those who consistently used CPAP therapy (at least 4 h per night, every day of the week) for a period ranging from 2 to 12 months showed significant enhancement in cognitive performance. In particular, these patients experienced an increase of 2.3 points in their MoCA scores (*p* < 0.001) and an increase of 1.2 points in their MMSE scores (*p* = 0.01) in comparison to those who did not adhere to the therapy [171]. These outcomes remained significant even after accounting for risk factors such as age, sex, BMI, initial sleepiness levels, and other health conditions. The study noted improvements in specific areas of cognition, such as visuospatial and executive functions, with a 1.0-point improvement in scores for these functions (*p* = 0.004) [171].

Despite the well-established role of OSA in the pathogenesis of several cardiovascular and cerebrovascular diseases, the benefit of positive airway pressure as a first-line therapy has shown inconsistent and sometimes insignificant benefits [108,172,173]. However, the lack of benefit or inconsistent results could be due to sample size in some studies, but compliance and adherence could also play a role [174]. Studies have shown that the level of compliance for CPAP has primarily been influenced by two factors: (1) severity of OSA, and (2) patients experiencing difficulties such as machine toleration and mask discomfort [174].

A year-long study was performed to understand the neurocognitive benefits of CPAP adherence interventional training on cognitively impaired older adults with mild OSA [175]. In this study, participants with mild OSA (AHI = 10–14) and amnestic mild cognitive impairment (MCI) were split into two groups: (A) CPAP adherent group (CPAP adherence ≥ 4 h per night for one year) and (B) CPAP non-adherent group. The CPAP adherent group received CPAP adherence interventional training for one year, and all participants completed tests on memory (Hodgkins Verbal Learning Test) and global cognition (Montreal Cognitive Assessment) at their 1-year follow-up. Study results showed that compared to the non-adherent group, the CPAP adherent group had significantly increased cognition scores and an eight-fold improvement in odds ratios for clinical dementia rating (CDR) scores. One limitation of this study is the small sample size, but the study remains an early study of clinical importance and a keystone trial for future research [175].

Another clinical trial by Richards and colleagues focused on the association between one year of CPAP adherence and slower cognitive decline in older OSA patients [176]. In this study, participants with MCI and mild OSA (AHI ≥ 10) were randomly placed into an MCI-CPAP adherent group or an MCI-CPAP non-adherent group and completed tests on psychomotor/cognitive processing speeds (Wechsler Adult Intelligence Scale Substitution Test) and memory (Hopkins Verbal Learning Test) after one year. The study reported that one year of CPAP adherence was significantly associated with improved memory scores and cognitive processing speeds [176]. These studies’ results showed that long-term adherence to CPAP treatment could have neurocognitive benefits that delay or prevent the progression of dementia. However, more long-term clinical research is needed to determine the extent of such therapeutic impact.

## 7. Future Directions

Neurocognitive degeneration is prevalent with increased age, with Alzheimer’s disease accounting for over 70% of cases. More clinical studies are needed to unravel the complex link between sleep and neurodegenerative processes, particularly how sleep disturbances contribute to the development of CSVD, amyloid angiopathy, and impaired clearance of neurotoxic waste. Longitudinal studies should also focus on how changes in sleep patterns across the lifespan affect cognitive decline and neurodegeneration, helping to establish causal relationships and mechanisms by which poor sleep accelerates brain aging. Integrating wearable technology into research can provide comprehensive, real-time data on sleep characteristics, offering more profound insights into their effect on brain health. The results of such studies could lead to novel therapeutic targets for preventing neurodegeneration.

Recent progress in antibody therapy for Alzheimer’s disease has energized research in neurocognitive degeneration. We hope these recent advances will result in carefully designed clinical trials for more targeted treatments and advancements in preventive protocols such as heart-brain cardiovascular imaging. These imaging protocols could serve as diagnostic tools to identify early markers of cardiocerebrovascular disease. Further studies are needed to fully understand the impact of findings from such imaging protocols on long-term neurocognitive outcomes.

## 8. Conclusions

OSA is a major cause of cerebrovascular disease, including neurocognitive decline. There is strong evidence showing that:

1. OSA is an independent risk factor for neurocognitive abnormalities.
2. OSA, hypertension, and arterial stiffness can create a triple-hit effect that contributes to the development of neurocognitive abnormalities.
3. A heart-brain imaging protocol could identify markers of early signs of pathology in both organ systems.

Addressing the hemodynamic contributions of OSA may reduce the risk of neurocognitive abnormalities, such as dementia, by preventing CSVD. Despite the significant cardiovascular implications of CSVD and its role in neurocognitive impairment, there are no specific treatments apart from managing diabetes, blood pressure, and sleep-disordered breathing. Current evidence and treatment strategies suggest that an early emphasis on these preventative measures might be beneficial in preventing dementia.

## Figures and Tables

**Table 1:** Effect of risk factors associated with OSA and cognitive decline—shown are hazard ratios for various risk factors and the development of neurocognitive abnormalities. There is a causal relationship between specific risk factors and sleep disturbances, such as REM disruption seen in OSA and the risk of neurocognitive abnormalities. Risk factors include sex, age, obesity, inflammation, and genetics.

	Diabetes	Inflammation	Obesity	Genetics	Age	Gender	Smoking & Alcohol
A	T2D in OSA 1.5 (1.2–2.0) [57]	CRP in OSA 0.58 (0.42–0.73) [61]	10% Weight gain 6.0 (2.2–17) [63]	TNF-α in OSA 1.82 (1.26–2.61) [66]	Age < 60: 35.0 (33.3–36.8)	NDD in women with AHI ≥ 15 3.4 (1.4–8.1) [71]	Alcohol in OSA 1.33 (1.10–1.62) [78]
B	OSA in T2D 1.48 (1.42–1.55) [58]	CRP in OSA 1.77 (1.28–2.26) [55]	BMI > 30 in OSA Male: 18 (14–23)	APOE variant in OSA 1.41 (1.06–1.87) [67]	Age > 60: 67.6 (65.2–70.0)	NDD in Women with APOE: 4.6 (1.0–20.7) [71]	Alcohol in OSA 2.03 (1.30–3.17) [73]
C	T2D + APOE 1.48 (1.36–1.60) [59]	TNF-α in NDD 2.4(0.3–4.5) [62]	BMI > 30 in OSA Female: 5.6(3.4–9.2) [64]	APOE in NDD [68] EA: 4.54 (3.99–5.17)	10-yr age increase, AHI ≥ 15 [69] Male: 1.94 (1.66–2.27)	Early menopause: 1.22 (1.11–1.34)	Smoking in OSA 2.5 (1.3–4.7) [74]
D	T2D onset in NDD 2.12 (1.50–3.0) [60]	IM + MetS in NDD 1.66 (1.19–2.32) [50]	PAF in NDD Obesity: 20.9 (13–28.8) [65]	White: 3.46 (3.27–3.65)	10-yr age increase, AHI ≥ 15 [69] Female: 3.20 (2.42–4.25)	Suppressed Androgen: 1.18 (1.08–1.29)	Alcohol in NDD, >14 drinks D.17.0 (4.0–32.0) [75]
E			NDD in Obesity w/o MetS before age 60 1.69 (1.16–2.45) [53]	Black: 2.18 (1.90–2.49)	NDD in Age + OSA [70] Age < 60: 3.30 (1.68–6.48)	Gender in NDD incidence [72] Female: 16.4 (15.2–17.6)	Alcohol in NDD, >14 drinks 1.72 (0.87–3.40) [76]
F				Hispanic: 1.90 (1.65–2.18)	Age > 60: 2.40 (1.65–3.48)	Gender in NDD incidence [72] Male: 12.3 (11.1–13.5)	Smoking in NDD 2.14 (1.65–2.78) [77]

Abbreviations: CI = confidence interval, T2D = type 2 diabetes; OSA = obstructive sleep apnea, APOE = apolipoprotein e, NDD = neurodegenerative disorder, CRP = c-reactive protein, TNF-α = tumor necrosis factor-alpha, IM = inflammatory markers, MetS = metabolic syndrome, PAF = population attributable fraction, AHI = apnea-hypopnea index, w/o = without, BMI = body mass index.
